# A self-repair history: compensatory effect of a *de novo* variant on the *FANCA* c.2778+83C>G splicing mutation

**DOI:** 10.3389/fgene.2023.1209138

**Published:** 2023-07-20

**Authors:** Ilaria Persico, Giorgia Fontana, Michela Faleschini, Melania Eva Zanchetta, Daniele Ammeti, Enrico Cappelli, Fabio Corsolini, Clara Mosa, Angela Guarina, Massimo Bogliolo, Jordi Surrallés, Carlo Dufour, Piero Farruggia, Anna Savoia, Roberta Bottega

**Affiliations:** ^1^ Department of Medical Sciences, University of Trieste, Trieste, Italy; ^2^ Department of Genetics and Microbiology, Universitat Autònoma de Barcelona, Barcelona, Spain; ^3^ Genomic Instability DNA Repair Syndromes Group, Joint Research Unit in Genomic Medicine UAB-IR Sant Pau, Sant Pau Biomedical Research Institute (IIB Sant Pau), Barcelona, Spain; ^4^ Institute for Maternal and Child Health—IRCCS “Burlo Garofolo”, Trieste, Italy; ^5^ Hematology Unit, IRCCS Istituto “G. Gaslini”—Genoa, Genova, Italy; ^6^ LABSIEM—Laboratory for the Study of Inborn Errors of Metabolism—Pediatric Clinic and Endocrinology—IRCCS Istituto “G. Gaslini”—Genoa, Genova, Italy; ^7^ Pediatric Onco-Hematology, ARNAS Civico Hospital, Palermo, Italy; ^8^ Centro de Investigación Biomédica en Red de Enfermedades Raras (CIBERER), Carlos III Health Institute, Madrid, Spain; ^9^ Department of Neurosciences, Biomedicine and Movement Sciences, University of Verona, Verona, Italy

**Keywords:** Fanconi anemia, somatic mosaicism, splicing mutation, *de novo* variant, natural gene therapy

## Abstract

**Introduction:** Fanconi anemia (FA) is a genome instability condition that drives somatic mosaicism in up to 25% of all patients, a phenomenon now acknowledged as a good prognostic factor. Herein, we describe the case of P1, a FA proband carrying a splicing variant, molecularly compensated by a *de novo* insertion.

**Methods and Results:** Targeted next-generation sequencing on P1’s peripheral blood DNA detected the known *FANCA* c.2778 + 83C > G intronic mutation and suggested the presence of a large deletion on the other allele, which was then assessed by MLPA and RT-PCR. To determine the c.2778 + 83C > G splicing effect, we performed a RT-PCR on P1’s lymphoblastoid cell line (LCL) and on the LCL of another patient (P2) carrying the same variant. Although we confirmed the expected alternative spliced form with a partial intronic retention in P2, we detected no aberrant products in P1’s sample. Sequencing of P1’s LCL DNA allowed identification of the *de novo* c.2778 + 86insT variant, predicted to compensate 2778 + 83C > G impact. Albeit not found in P1’s bone marrow (BM) DNA, c.2778 + 86insT was detected in a second P1’s LCL established afterward, suggesting its occurrence at a low level *in vivo*. Minigene assay recapitulated the c.2778 + 83C > G effect on splicing and the compensatory role of c.2778 + 86insT in re-establishing the physiological mechanism. Accordingly, P1’s LCL under mitomycin C selection preserved the FA pathway activity in terms of FANCD2 monoubiquitination and cell survival.

**Discussion:** Our findings prove the role of c.2778 + 86insT as a second-site variant capable of rescuing c.2778 + 83C > G pathogenicity *in vitro*, which might contribute to a slow hematopoietic deterioration and a mild hematologic evolution.

## Introduction

Fanconi anemia (FA) is a rare genetic syndrome due to mutations in one of at least 22 genes, whose encoded proteins exert a consolidate role in genome integrity maintenance. Upon interstrand crosslinks (ICLs) impairing replication-fork progression, a nuclear complex of seven FA proteins (FANCore) activates FANCD2 through monoubiquitination, allowing its binding to the lesion and recruiting further players for DNA repair (Kottemann and Agata, 2013; Bogliolo and Surrallés, 2015). Consistently, FA cells show a distinctive hypersensitivity to ICL-inducing drugs (e.g., mitomycin C, MMC, and diepoxybutane, DEB), resulting in increased chromosome fragility, a cellular marker that has been deeply exploited to develop accurate diagnostic tests (e.g., DEB test) (Auerbach, 1988; Auerbach, 2003).

Genetic instability is responsible for the main clinical features of FA, such as bone marrow (BM) failure, within the first decade of life in 80% of cases, and heightened predisposition to hematological and solid malignancies with a 30% and 40% incidence in the adulthood, respectively (Kutler, 2003; Alter et al., 2018). Nevertheless, the higher rate of error underlying the disorder could also elicit genetic events (e.g., gene conversion, intragenic crossover, back mutations, and second-site mutations) with the potential to restore a wild-type (WT) allele and, thus, lead to a phenotypic correction both in terms of DNA repair and ICL-inducing agent resilience (Lo Ten Foe et al., 1997; Waisfisz et al., 1999; Gregory et al., 2001).

Somatic mosaicism, indeed, occurs in a considerable subset of FA subjects, estimated at around 15%–25% of all cases (Lo Ten Foe et al., 1997; Soulier et al., 2005; Ramírez et al., 2021). It may arise from reversion or compensatory mutations in either pluripotent or committed hematopoietic stem and progenitor cells (HSPCs) (Gregory et al., 2001; Gross et al., 2002; Soulier et al., 2005; Nicoletti et al., 2010). Accordingly, it has been described in blood and/or BM cells, and also in fibroblasts in a single case (Asur et al., 2017).

In the standard clinical routine, FA mosaicism is first recognized via the DEB test on cultured T lymphocytes, unveiling a population resistant to clastogenic concentrations typically toxic to FA cells (Lo Ten Foe et al., 1997; Castella et al., 2011; Fargo et al., 2014). Optimized algorithms are now available to diagnose the condition (Soulier et al., 2005; Kalb et al., 2007; Ramírez et al., 2021), but evaluations of refractory cell percentage within a single lineage (i.e., T lymphocytes) and timepoint may not be representative of multilineage hematopoietic reversion (Gross et al., 2002). In view of this, genetic sequencing, assessment of distinct blood and BM progenitor populations (e.g., BM-derived colony forming cells, CFCs), and sensitivity to ICL-inducing drugs in other cells (i.e., fibroblasts) are regarded as valuable tools to confirm FA mosaicism, which, however, are still confined to the research dimension due to their difficult adaptation to the medical routine (Gregory et al., 2001).

Within this framework, we present a young FA patient carrying a *FANCA* allele characterized by a splicing variant, whose deleterious effect is corrected by a *de novo* mutation event at least *in vitro*.

## Materials and methods

### Family study and clinical features

The propositus was diagnosed with FA at the age of 7 based on the positive chromosomal DEB test. The subject presented mild and isolated thrombocytopenia since 1.5 years of age, and at the time of diagnosis, his peripheral blood (PB) smear revealed white blood cells (WBCs) 6.7 × 10^9^/l, neutrophils 2.5 × 10^9^/l, hemoglobin (Hb) 13.3 g/dL, mean corpuscular volume (MCV) 95 fL, and platelets 113 × 10^9^/l (Table 1). Hematopoietic cellularity on BM trephine was 40%, and no dysplastic features were visible on BM cellular morphology. Moreover, chromosomal analysis showed a normal karyotype (46, XY) of BM cells. P1 was in the 10th percentile for height and weight, and was investigated through abdominal ultrasound, echocardiography, thyroid ultrasound, brain magnetic resonance imaging, audiometry, and spirometry, without appreciating any malformation or abnormality. Both bone age and density scan were in the normal range as well.

**TABLE 1:** Monitoring of the proband (P1)’s clinical features. The table illustrates the clinical data on P1’s peripheral blood (PB) smear and bone marrow (BM) analysis at the ages of 7 (time of diagnosis) and 11 (follow-up).

	P1’s age	
	7 years old (diagnosis)	11 years old (follow-up)
Peripheral blood (PB) smear		
White blood cells (WBCs) (x10^9/L)	6.7	4.6
Neutrophils (x10^9/L)	2.5	1.2
Hemoglobin (Hb) (g/dL)	13.3	12.3
Mean corpuscular volume (MCV) (fL)	95	103
Platelets (x10^9/L)	113	50
Bone marrow (BM)		
Hematopoietic cellularity (%)	40	29
Karyotype	46, XY	46, XY

The patient entered the monitoring plan in use at the IRCCS Istituto “G. Gaslini” (Genoa, Italy). Over follow-up, he showed a moderate decline of the overall blood count, especially affecting the platelet population. Currently, 5 years after diagnosis and at the age of 11, WBCs are 4.6 × 10^9^/l, neutrophils 1.2 × 10^9^/l, Hb 12.3 g/dL, MCV 103 fL, and platelets 50 × 10^9^/l (Table 1). Hematopoietic cellularity on BM trephine is 25%–28%, and BM cells still have a normal karyotype without dysplastic signs. Based on this finding, although hematopoietic stem cell (HSC) donor search was initiated either in the family or in the international registries, HSC has not been planned yet. The research was approved by the Ethics Review Boards at the institutions that enrolled the patients. According to the Declaration of Helsinki, all the family members or their legal guardians signed written informed consent for the analysis.

### Cell cultures

P1’s lymphoblastoid cell lines (LCLs) were generated from primary lymphocytes isolated from peripheral blood (Bottega et al., 2018). LCLs and HEK293 cells were grown in RPMI 1640 1X (L-glutamine) and DMEM 1X (Euroclone, Pero, IT) media, respectively, with 10% fetal bovine serum (FBS) and 1% penicillin–streptomycin at 37°C in a humidified atmosphere containing 5% CO_2_. Following DNA, RNA, or protein extraction, cell cultures were transferred to RPMI or DMEM with 10% DMSO and then frozen in liquid nitrogen for storage.

### Mutation screening

Genomic DNA was extracted from patient’s peripheral blood and LCLs. FA genes were analyzed using the Ion PGM system for next-generation sequencing (Life Technologies, Carlsbad, CA), as described in De Rocco et al. (2014). *FANCA* deletion was identified analyzing Ion PGM row data, as described in Nicchia et al. (2015). For Sanger sequencing, PCR was carried out using the KAPA2G Fast HotStart ReadyMix (KAPA Biosystems, Wilmington, MA) and the primer pair 28F 5′-GTT​GAT​GGT​CTG​TTT​CCA​CC-3′ and 28R 5′-ACC​CTA​GAC​TCG​AGA​CGA-3′ for the detection of c.2778 + 83C > G and c.2778 + 112T > C variants. PCR products were purified with ExoSAP-IT (Applied Biosystems, Foster City, CA) and sequenced using the ABI PRISM sequencer (Applied Biosystems, Foster City, CA). Nucleotide numbering reflects the *FANCA* cDNA with +1 corresponding to the A of the ATG translation initiation codon in the reference sequence (RefSeq NM_000135). Variants identified were searched in the following annotation databases: Single-Nucleotide Polymorphism Database (dbSNP; http://www.hgmd.cf.ac.uk/ac/index.php), Genome Aggregation Database (gnomAD; https://gnomad.broadinstitute.org), and Human Gene Mutation Database (HGMD; http://www.hgmd.cf.ac.uk/ac/index.php). The *in silico* analyses of splicing mutations were carried out using Splice Site Prediction by Neural Network (NNSplice; http://www.fruitfly.org/seq_tools/splice.html).

### RNA analysis

Total RNA was extracted from the patient’s LCLs and peripheral blood using the High Pure RNA Isolation Kit (Roche, Basel, CH) and PAXgene Blood RNA Kit IVD (QIAGEN, Hilden, DE), respectively, according to the manufacturer’s instructions. cDNA was synthetized using the Transcriptor First Strand cDNA Synthesis Kit (Roche, Basel, CH), and RT-PCRs were prepared with KAPA2G Fast HotStart (KAPA Biosystems, Wilmington, MA). To verify the deletion from exons 11 to 14, RT-PCR primers 9F 5′-TTG​ATG​TAC​TGC​AGA​GAA​TGC-3′ and 16R 5′-GTG​TCT​TGG​CCA​ATG​AGA​TG-3’ were used. To assess the c.2778 + 83C>G effect, RT-PCR primers 27F 5′-AGC​TGC​TTA​TCT​CCA​GGC​C-3, 28R_+83 5′-CAA​ACC​CTA​GAC​TCA​GGA​CG-3,′ and 30R 5′-GGA​AAT​CCA​TCA​GTG​CGT​TG-3′3′ were designed within the predicted intronic retention and used in Savino et al. (1997) to characterize the variant. RT-PCR amplicon extraction from 3% agarose gel was performed using the QIAquick Gel Extraction Kit (QIAGEN, Hilden, DE) in line with the manufacturer’s instructions. To increase the performance and purify the extraction, the products obtained were further subjected to classical PCR with the same primer pair used for RT-PCR. All RT-PCR amplicons were purified and sequenced, as reported previously.

### Plasmid construction and hybrid minigene assay

A 533-bp cassette that contains *FANCA* exon 28 with portions of introns 27 and 28 was created by the amplification of cDNA obtained by patients’ PB or LCLs in order to obtain all the investigated haplotypes. Amplification primers (clonF 5′-ATCATCCATATGGAATGTGGGGTTGTG-3′ and clonR 5′-TGTTGTCATATGGTCAAGATTCCAATC-3′) were designed with an NdeI restriction site (underlined) at the end in order to allow the cloning into the α-globin minigene under the control of the α-globin promoter and the SV40 enhancer (Mattioli et al., 2014).

HEK293 cells seeded in a six-well plate were transfected with 3 ug of each minigene plasmid using Lipofectamine 3000 (Thermo Fisher Scientific, Waltham, MA).

For the analysis of the splicing isoforms, the primers α2F (5′-CTT​CAA​GCT​CCT​AAG​CCA​CTG-3′) and β2R (5′-CAC​CAG​GAA​GTT​GGT​TAA​ATC-3′) were used.

### Western blot

Protein whole-cell extracts were prepared using RIPA buffer (Upstate Biotechnology, Lake Placid, NY). In brief, the cells were washed with phosphate-buffered saline and resuspended in RIPA buffer containing protease and phosphatase inhibitors (Roche, Basel, CH) and benzonase (Novagen, Madison, WI) at 25 U/mL. The samples were lysed and quantified with the Pierce BCA Protein Assay Kit (Thermo Fisher Scientific, Waltham, MA), according to the manufacturer’s instructions. Protein extracts were then loaded on 6% SDS-PAGE gel and blotted with the following antibodies: anti-FANCA (rabbit, A301-980A, Bethyl, 1:2000), anti-FANCD2 (rabbit, Abcam, ab2187; 1:2000), anti-vinculin (rabbit, Abcam, ab155120; 1:5000) as the loading control, and anti-rabbit (goat, Bethyl, A120-101P, 1:2500). Immunodetection was operated with Immobilon Crescendo Western HRP Substrate (Merck Millipore, Burlington, MA) and Pierce ECL Western blotting Substrate (Thermo Fisher Scientific, Waltham, MA) to reveal FANCA, and FANCD2 and vinculin proteins, respectively. The densitometric analysis of the anti-FANCA antibody signal was performed via ImageJ software, based on the use of vinculin for data normalization and the comparison with both positive and negative controls’ results. To analyze FANCD2 monoubiquitination, the cells were treated with 2 mM hydroxyurea (HU) (Sigma-Aldrich, St. Louis, MO) for 18 h prior to sample collection.

### Mitomycin C (MMC) survival assay

LCL survival under MMC treatment (0, 3, 10, 33, 100, and 333 nM) was tested after a few cell passages (Time 0) and a month after the establishment of the cultures (Time 1), as previously described in Bottega et al. (2019).

## Results

### Mutation screening

**FIGURE 1:**
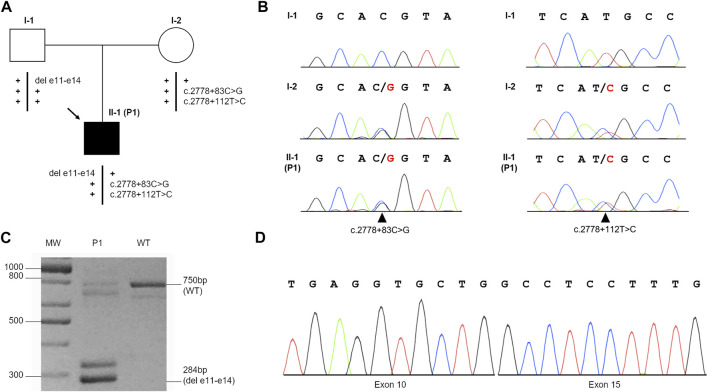
Identification of c.2778 + 83C>G and c.2778 + 112T>C variants and the deletion of exons 11–14 (del e11-e14) in the *FANCA* gene. **(A)** Patient’s familial pedigree: the black arrow shows the proband (II-1, P1), and plus symbols (+) represent the wild-type alleles. **(B)** Electropherograms of the family members’ variants confirmed by Sanger sequencing: black arrows indicate c.2778 + 83C>G (left) and c.2778 + 112T>C (right) substitutions in the heterozygous status. **(C)** RT-PCR performed with primers 9F and 16R on the mRNA of the proband (P1) and wild-type (WT) control. In addition to the expected fragment of 750 bp, P1 showed a 284-bp band hypothesized to be the deleted product. The other bands detected in the samples were likely to represent alternative splicing isoforms or non-specific products. **(D)** Sanger sequencing of the 284-bp product shows the junction of exon 10 with exon 15, as expected by MLPA analysis.

Targeted next-generation sequencing (t-NGS) of patient’s DNA from peripheral blood (PB) revealed two single-nucleotide heterozygous substitutions (c.2778 + 83C > G and c.2778 + 112T > C) in intron 28 of the *FANCA* gene, both confirmed in the proband (II-1, P1) and his mother (I-2) by Sanger sequencing, indicating they are *in cis* (Figures 1A, B). The c.2778 + 83C > G transversion is reported in GnomAD (“uncertain significance”, MAF: 0.000004012) and in HGMD (“disease causing”) and, according to NNSplice software, predicted to generate a strong cryptic donor splice site (GT) at position 84–85 of *FANCA* intron 28, favoring its recognition over the physiological splicing site (SS) (Table 2). The 2778 + 112T > C variant is annotated in GnomAD (MAF: 0.0002739) and dbSNP (rs140073727), and is likely not to affect splicing, not even when combined with c.2778 + 83C > G (Table 2). On the other allele, NGS data allowed us to postulate an intragenic deletion of *FANCA* of paternal origin encompassing exons 11–14 (Figure 1A), and subsequently confirmed by MLPA analysis (data not shown) and RT-PCR on total PB RNA (Figure 1C, D).

**TABLE 2:** Splicing scores calculated with NNSplice. The scores are reported both for the single FANCA variants identified in intron 28 and those combined in a specific haplotype, as found in FA patients.

FANCA NM_000135					Possible donor site (underlined)^a^		Splicing site score^b^
FANCA variants	Wild-type				WT site	*tgttcac* ***gt*** *aggtga*	**0.68**
					Cryptic site	*gcagcac* ***gt*** *aggtga*	0.47
	c.2778 + 83C>G				WT site	*tgttcac* ***gt*** *aggtga*	0.68
					Cryptic site	*gcagca* ***ggt*** *aggtga*	**0.96**
	c.2778 + 112T>C				WT site	*tgttcac* ***gt*** *aggtga*	**0.68**
					Cryptic site	*gcagcac* ***gt*** *aggtga*	0.47
	c.2778 + 40G>T				WT site	*tgttcac* ***gt*** *aggtga*	**0.68**
					Cryptic site	*gcagcac* ***gt*** *aggtga*	0.47
	c.2778 + 86insT				WT site	*tgttcac* ***gt*** *aggtga*	**0.68**
					Cryptic site	*gcagca* ***ggtt*** *aggtga*	**0.78**

Haplotype ID		Patient	Intron 28 position				
FANCA haplotype^c^	1 GCTT	WT’s LCL	+40	G	WT site	*tgttcac* ***gt*** *aggtga*	**0.68**
			+83	C			
			+86	T	Cryptic site	*gcagcac* ***gt*** *aggtga*	0.47
			+112	T			
	2 GGTC	P1’s PB P2’s LCL	+40	G	WT site	*tgttcac* ***gt*** *aggtga*	0.68
			+83	G			
			+86	T	Cryptic site	*gcagca* ***ggt*** *aggtga*	**0.96**
			+112	C			
	3 TGTC	P3’s LCL	+40	T	WT site	*tgttcac* ***gt*** *aggtga*	0.68
			+83	G			
			+86	T	Cryptic site	*gcagca* ***ggt*** *aggtga*	**0.96**
			+112	C			
	4 GGTTC	P1’s LCLs	+40	G	WT site	*tgttcac* ***gt*** *aggtga*	**0.68**
			+83	G			
			+86	TT	Cryptic site	*gcagca* ***ggtt*** *aggtga*	**0.78**
			+112	C			

^a^ The c.2778 + 83C>G and c.2778 + 86insT variants are indicated in bold red and bold green, respectively. Canonical and cryptic sites refer to c.2778 + 1_2778+2 GT and c.2778 + 84_2778 + 85GT, respectively.

^b^ The preferentially used site is colored.

^c^ Nucleotide changes with respect to the wild-type are indicated in red.

### Effect of c.2778 + 83C > G on RNA splicing

**FIGURE 2:**
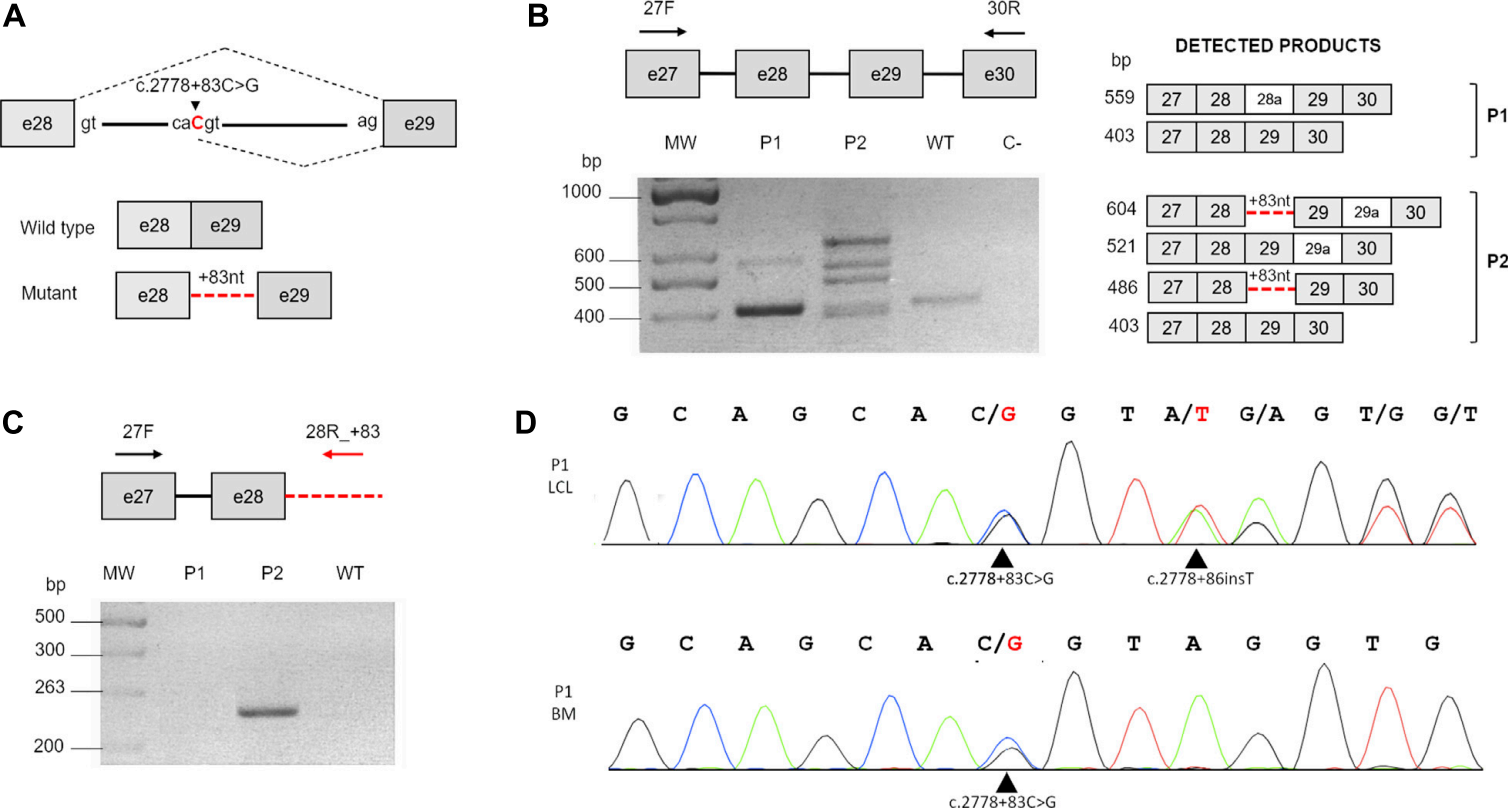
Analysis of c.2778 + 83C>G. **(A)** Schematic representation of the c.2778C>G splicing effect. Gray boxes indicate exons, and the continuous line indicates the intron. The nucleotide substitution is indicated in red. The black dotted lines represent the normal splicing pattern (above) and the aberrant effect on the process (below). **(B)** RT-PCR carried out with primers 27F and 30R (black arrows). The schematic representation on the right shows the expected RT-PCR products (dark gray boxes, exons; light gray boxes, alternatively spliced exons; and red dotted lines, inclusion of the first 83 nucleotides of intron 28). MW, molecular weight; WT, wild-type; C, negative control. **(C)** Comparison of the different splicing effects of the c.2778 + 83C>G transversion in patients P1 and P2. RT-PCR carried out with primers in exon 27 (27F) and within the 83-bp insertion of intron 28 (28R_+83) (red arrows) detectable only in P2. **(D)** Sequencing analysis of intron 28 using DNA from the first LCL established from P1 (P1 LCL) and his bone marrow cells (P1 BM). Black arrows indicate c.2778 + 83C>G in both DNA samples and the novel c.2778 + 86insT insertion detectable only in LCL.

To determine the c.2778 + 83C > G splicing effect, we performed RT-PCR on the total RNA from a LCL established from the proband (P1) (Figure 2A, B). As a control, we used the LCL RNA of another *FANCA* patient (P2), compound heterozygote for a large intragenic deletion (exons 11–31 deletion; data not shown) and the same c.2778 + 83C > G mutation (Savino et al., 1997), leading to an alternative transcript with the retention of the first 83 nucleotides of intron 28 (Figure 2A; Table 2). In line with the literature (Savino et al., 1997), in P2, we observed four bands. The fastest of 403 bp represents the wild-type band, suggesting the production of a small amount of normal transcript. The product of 486 bp (403 + 83) is due to retention of the first 83 nucleotides of intron 28. The remaining two are spliced products (521 bp and 604 bp) with an insertion of 118 bp (alternatively processed exon 29a with an unknown biological effect) between exons 29 and 30 (Figure 2B).

In P1, instead, we revealed only two bands corresponding to the WT amplicon (403 bp) and a novel faint product (559 bp) not detectable in P2 and in the control (Figure 2B), later identified by Sanger sequencing as another alternative spliced form of *FANCA* with 156 additional bp (another alternatively cleaved exon 28a with uncertain biological significance) between exons 28 and 29. Of note, the analysis did not reveal any product with the partial intronic retention. To further confirm these data, we carried out a RT-PCR using a reverse primer specifically designed to anneal over the 83-bp insertion of intron 28 (Figure 2C). As in the previous analysis, the expected amplicon (263 bp) was detected in only P2’s sample (Figure 2C), suggesting a different c.2778 + 83C > G splicing impact on P1.

In trying to elucidate whether any mutational event could have restored the physiological splicing pattern in P1, we sequenced exon 28 and its flanking regions from the genomic DNA of both patients’ cultured cells. However, in P2, we ascertained the c.2778 + 83C>G mutation and the 2778 + 112T>C variant, both in an homozygous condition, and therefore, on the same haplotype, in P1, we unveiled a third alteration, c.2778 + 86insT, a *de novo* thymine insertion (Figure 2D) not found in the genomic DNA from neither the patient’s PB and BM cells nor from his parents’ PB (data not shown). We confirmed the presence of c.2778 + 86insT also in a second P1’s LCL established 2 years apart (data not shown), suggesting its onset *in vivo*.

### Compensatory effect of c.2778 + 86insT on splicing machinery

**FIGURE 3:**
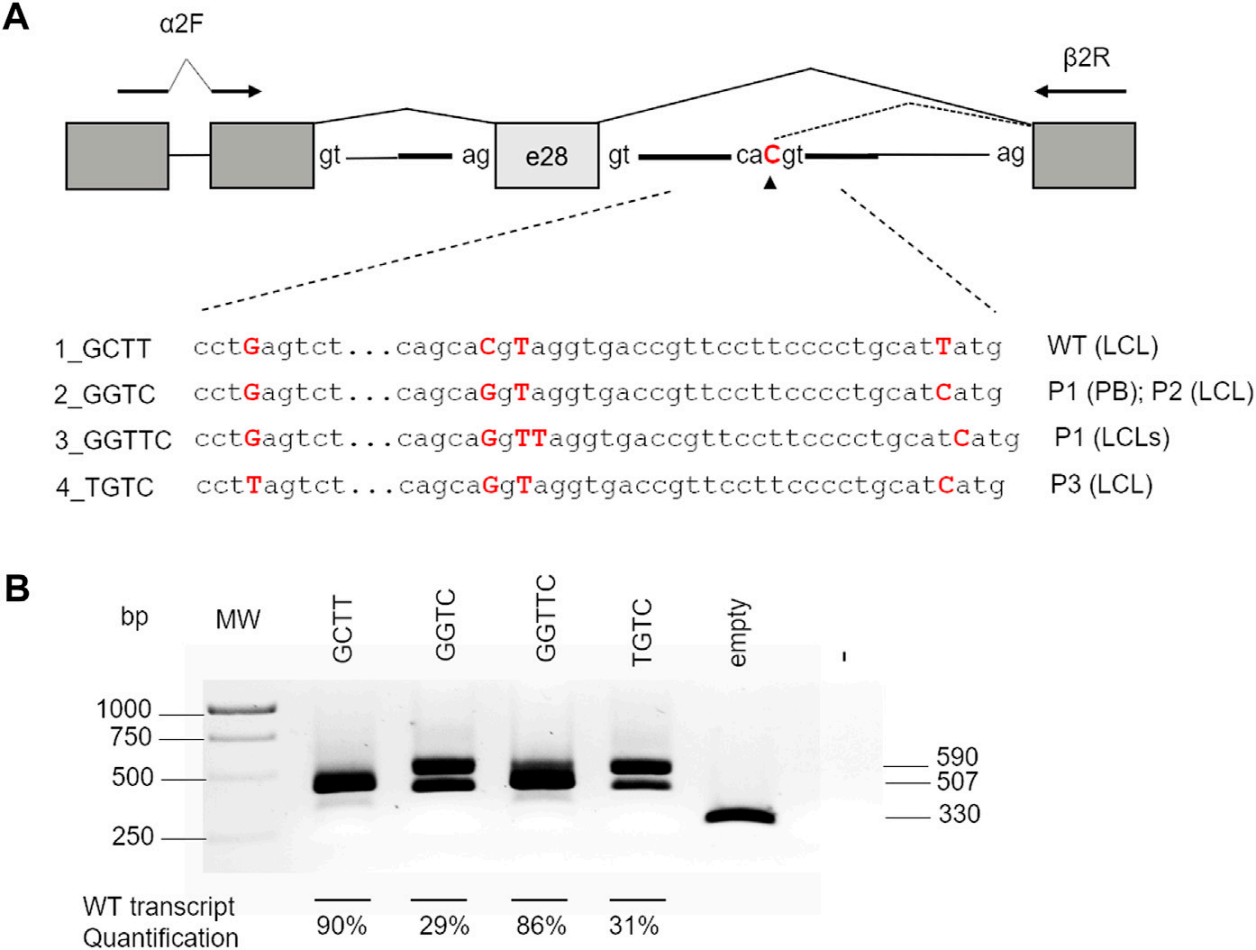
Effect of different haplotypes on splicing. **(A)** Schematic representation of the pTBNdeI minigene containing exon 28 and the intronic flanking regions with the different haplotypes analyzed. Boxes represent exons, and arrows indicate the primers used for PCR analysis. The nucleotides generating the different haplotypes cloned into the vector are indicated in bold red. **(B)** RT-PCR products of 507 bp (correct removal in intron 28) and 590 bp (retention of the first 83 bp of intron 28) obtained after transfection splicing of the different constructs in HEK293 cells. The percentages of the WT product are indicated as the mean ± standard deviation of at least three independent experiments. MW, molecular weight.

According to NNSplice software, the combination of c.2778 + 83C>G and c.2778 + 86insT on the same allele was predicted to decrease the strength of the cryptic splice site at the intronic position 83 (Table 2). Thus, to further investigate the contribution of c.2778 + 83C>, c.2778 + 112T>C, and c.2778 + 86insT variants on pre-mRNA processing, we generated a pTBNdeI hybrid minigene, where we subcloned *FANCA* exon 28 with adjacent portions of intron 27 and the WT or mutagenized forms of intron 28 (Pagani et al., 2003) (Figure 3). Owing to the amplification of the genomic DNA from patients’ PB and/or LCL for the creation of the *FANCA* cassette, the analysis allowed us to ascertain the cis arrangement of c.2778 + 83C>G and c.2778 + 86insT in P1, as determined by Sanger sequencing on the subcloned fragments. We also examined the effect of c.2778 + 40G>T, an unreported, apparently neutral *de novo* variant, found in heterozygosis in a *FANCA* patient (P3) carrying c.2778 + 83C>G and c.2778 + 112T>C in the homozygous status (data not published). Minigenes expressing the four different haplotypes of intron 28 (Figure 3; Table 2) were transiently transfected in HEK293 cells, and the pattern of splicing was analyzed by RT-PCR amplification with specific primers. Transfections confirmed the expression of two isoforms corresponding to the transcripts generated by the competition of the physiological (TCACgtaggt; 507 bp) and cryptic (gcacgtaggt; 590 bp) donor splice sites at different extents. Indeed, the wild-type GCTT haplotype was mainly associated with normal splice processing, with intron 28 removal in almost 90% of transcripts (Figure 3). On the contrary, analysis of the GGTC (P1’s PB and P2’s LCL) and TGTC (P3’s LCL, differing at position c.2778 + 40) mutant haplotypes showed a significant increase (almost 70%) in the splicing isoform with the 83-bp intronic retention due to the strong recognition of the cryptic splice site (gcaGgtaggt). The identical pattern shared by the two aberrant haplotypes GGTC and TGTC indicated that the c.2778 + 40G > T variant did not affect the alternative splicing process. Interestingly, the GGTTC haplotype (P1’s LCL), carrying c.2778 + 86insT, restored almost completely the splicing of intron 28, providing an explanation to the RT-PCR data observed in P1’s LCL (Figure 2C).

### c.2778 + 86insT restores the cellular FA phenotype

**FIGURE 4:**
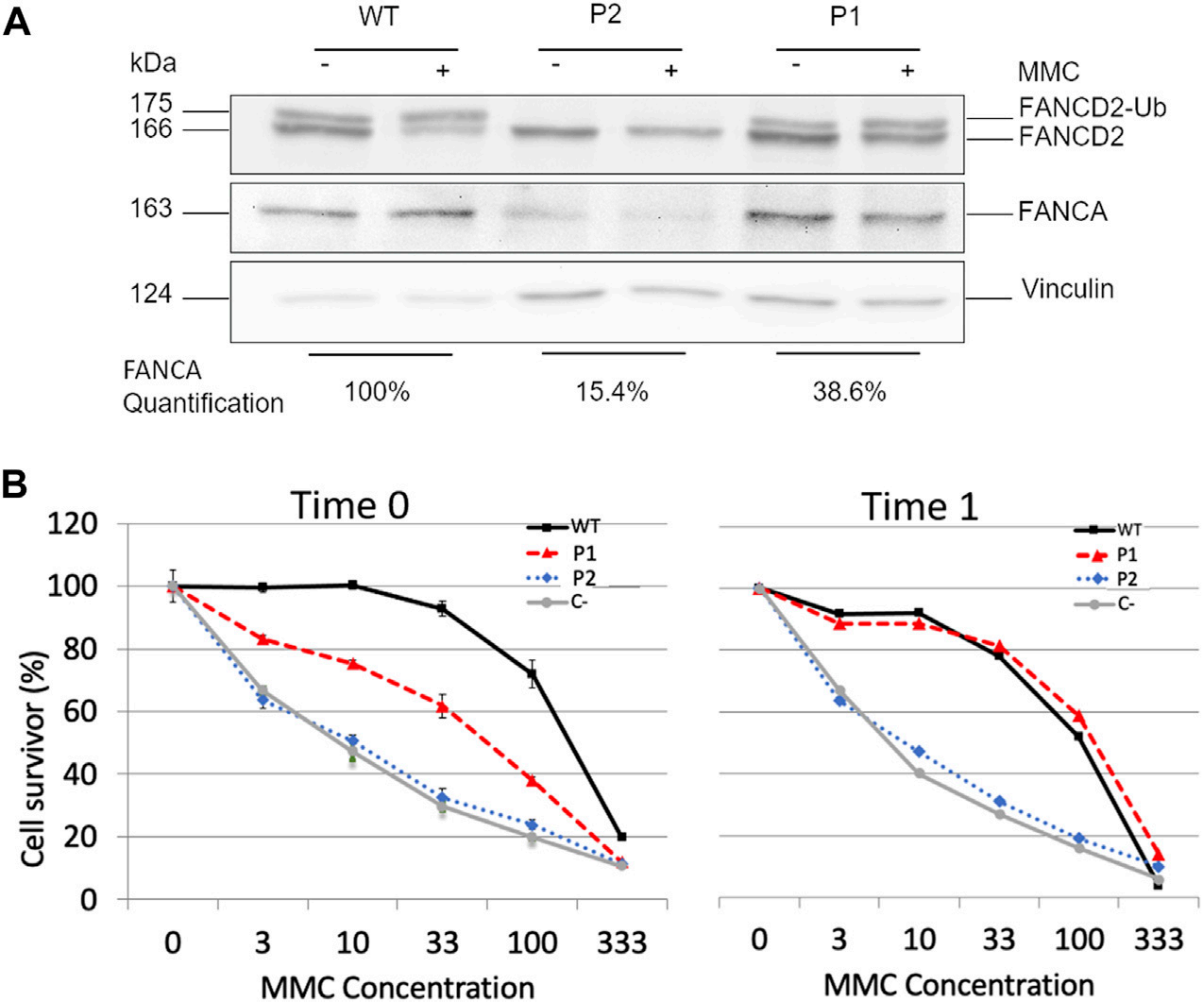
Effects of c.2778 + 86insT in P1′ LCLs. **(A)** Evaluation of FANCA expression and FANCD2 monoubiquitination. Western blotting (WB) of FANCA shows a reduction of the expression level in both P1 and P2 with the control (WT), albeit at different extents (15.4% and 38.6% in P2 and P1, respectively). WB of FANCD2 in the absence (−) or presence (+) of hydroxyurea (HU) reveals that only in P1, there is a detectable level of monoubiquitinated FANCD2 (FANCD2-Ub). Vinculin was used as the loading control. **(B)** Cell survival of LCL treated with MMC. Cells from P1, P2, and wild-type were tested after a few cell passages (Time 0) and after 1 month of culture under stress conditions (Time 1). C- represents cells of a FANCA−/− patient used as negative control. Although no significant differences were detected in P2 overtime, the cells of P1 manifested a transition from an intermediate sensibility between WT and C- to complete phenotypic reversion. Data are reported as the mean ± standard deviation of three independent experiments.

In order to confirm the compensatory effect of c.2778 + 86insT, we compared P1 and P2’s LCL cellular phenotypes. Since both the affected individuals were compound heterozygous for c.2773 + 83C > G and a large *FANCA* deletion of exons 11–14 in P1 and exons 11–31 in P2, only the allele with the intronic mutation was expected to contribute to any protein production. We detected FANCA protein in both P1 and P2’s LCLs, albeit at lower levels in comparison to the WT control (Figure 4A). Indeed, the densitometric analysis of immunoblot bands allowed us to assess a reduction to 38.6% and a relevant decrease to 15.4% of protein expression in P1 and P2, respectively (Figure 4A). These findings were consistent with the role of c.2778 + 86insT in restoring, even partially, the activity of the physiological donor splice site.

Subsequently, we evaluated whether the expression level of FANCA could guarantee FANCD2 monoubiquitination. We observed detectable levels of monoubiquitinated protein in both untreated and treated cells with HU in P1 but not in P2 (Figure 4A), suggesting that about 40% of FANCA was enough to preserve this post-translational modification. Conversely, when FANCA expression was significantly reduced as in P2 (Figure 4A), no FANCD2 monoubiquitination was detected.

Assuming that P1’s cells used to establish the LCL were a mosaic for the c.2778 + 86insT variant and those carrying it could have some proliferative advantages, we eventually examined the proficiency of patients’ LCLs to survive different concentrations of MMC (3–100 nM) (Figure 4B). We, thus, thawed one of the original vials stored after immortalization and carried out the test after a few cell passages (Time 0) and 1 month of culture (Time 1). P2’s cells showed a MMC sensitivity comparable to that of the FANCA−/− control at both time points. On the contrary, P1’s LCL had a survival rate intermediate between that of WT and FANCA−/− cells at Time 0, and a completely restored phenotype at Time 1 (Figure 4B), further corroborating the compensatory role of c.2778 + 86insT.

## Discussion

Hematopoietic mosaicism, showing an incidence of 25% in FA, is a phenomenon associated with phenotypic reversion at least in cells and is regarded as a natural gene therapy since its first identification (Youssoufian, 1996). The case described (P1) clearly illustrates the direct involvement of a *de novo* compensatory variant in rescuing a *FANCA* mutant allele, otherwise known for its deleterious effect in FA, at the molecular level. P1 is compound heterozygous for a large intragenic deletion and the c.2778 + 83C > G transversion of *FANCA*. The latter variant has been identified in linkage disequilibrium with c.2778 + 112T > C in another two apparently unrelated patients (namely, P2 (Savino et al., 1997) and P3 in this paper). Our hypothesis is that c.2778 + 83C > G mutation occurred on a haplotype carrying the c.2778 + 112T > C variant, suggesting a possible founder effect of this mutant haplotype in the Italian population.

The c.2778 + 83C > G variant is located within a cryptic, almost “silent,” donor splice site in intron 28, whose score significantly increases in the presence of this substitution, suggesting a potential impact of c.2778 + 83C > G on the splicing machinery. In line with Savino et al. (1997), we recapitulated the c.2778 + 83C > G splicing effect in LCL of P2, which triggered the activation of the cryptic donor splice site, resulting in an alternative cleavage with the retention of the first 83 intronic nucleotides. On the contrary, we did not detect any aberrant splicing in P1’s LCL, leading us to investigate the reason of this unexpected result.

In addition to c.2778 + 83C > G and c.2778 + 112T > C, the sequencing of P1’s LCL DNA identified a single T insertion at the intronic position 86 (c.2778 + 86insT), which was regarded as a *de novo* mutation since not being detected in parent’s PB. Bioinformatics analysis predicted c.2778 + 86insT to significantly reduce the strength of the mutant cryptic donor site and restore the activity of the physiological donor site, an effect that we later confirmed using a minigene model. Consistently, when compared to P2’s LCL, in P1, we observed higher expression levels of FANCA, which, albeit under 50%, sustained FANCD2 monoubiquitination and the WT survival rate under MMC treatment, indicating a proliferative advantage of c.2778 + 86insT reverted clones at least *in vitro*. Moreover, consistent with the recognition of the physiological donor site, FANCA, although at a low level, is detectable in the LCL of P2. Although these cells do not monoubiquitinate FANCD2 and are significantly sensitive to cross-linking agents at least in our experimental procedures, we cannot exclude that FANCA retains some residual activity and that c.2778 + 83C > G might be regarded as an hypomorphic germline mutation.

The *de novo* c.2778 + 86insT event is likely to have arisen *in vivo* as it was detected in two LCLs established two years apart. Nevertheless, the analysis of the genomic DNA from either proband’s PB or BM cells did not allow detecting the *de novo* T insertion, suggesting that the number of cells with this mutation is very limited. Moreover, the lack of suitable BM material prevented us from searching the variant in different cell populations and defines the stage of the hematopoietic hierarchy in which the mutational event occurred.

At diagnosis, P1 had a mild somatic phenotype and very mild hematological involvement, which are consistent with a beneficial effect of the *de novo* c.2778 + 86insT variant. Indeed, even if over time, the hematopoietic function showed some decline (mainly expressed by the drop of the platelet count from 113 × 10/^9^ to 50 × 10/^9^ after a 5-year follow-up), this decrement was less rapid than that observed in FA patients (median age at HSC transplantation is about 8 years) (Mehta et al., 2017) and, consistently, HSC transplantation has not been planned in the patient yet.

Considering that the FA phenotype is characterized by extensive variable clinical expression, even in patients carrying the same mutations, we cannot propose any correlation between the relative mild phenotype of the proband with the FANCA alterations identified. However, we could speculate that they contribute to the relatively stable blood counts, diminished or delayed need for HSC transplant, and prolonged survival through the combined effect of a potential hypomorphic germline mutation (c.2778 + 83C > G) and a *de novo* variant (c.2778 + 86insT) that restores the FA cellular phenotype at least *in vitro*. Further follow-up will monitor the evolution of the disease in terms of the marrow function, from slow deterioration, stabilization, or even improvement.

Nowadays, HSCT is the only curative approach for FA hematological defects, and according to the current guidelines, it should be performed when the marrow function declines below the level of transfusion dependence or in case of evolution to myelodysplastic/acute myeloid leukemia (MSD/AML) (Dufour, 2017). Despite the unquestionable progress in the last three decades, this procedure is still associated with important side effects, namely, transplant-related mortality and an increased risk of post-transplant malignancies (Rosenberg et al., 2005; Anur et al., 2016; Kutler et al., 2016; Alter et al., 2018). In this context, increasing the sensitivity of molecular testing for the identification of genetic events with a possible positive effect may delay or even inhibit the usage of HSCT, containing the risks associated with this gold standard procedure, and improving patients’ care. The characterization of the potential “natural” gene therapy effect of c.2778 + 86insT or similar events would indeed be critical for the development of novel personalized therapeutic strategies (e.g., genome editing with the CRISPR/Cas9 system and synthesis of novel drugs acting on splicing), enabling more accurate treatment and decision plans toward the sizable subset of FA mosaic subjects.

## Data Availability

The original contributions presented in the study are publicly available. This data can be found here: https://www.ncbi.nlm.nih.gov/bioproject/PRJNA1013760.
