# Erratum: A self-repair history: compensatory effect of a *de novo* variant on the FANCA c.2778+83C>G splicing mutation

**DOI:** 10.3389/fgene.2023.1293778

**Published:** 2023-09-20

**Authors:** Frontiers Production Office

**Affiliations:** Frontiers Media SA, Lausanne, Switzerland

**Keywords:** Fanconi anemia, somatic mosaicism, splicing mutation, *de novo* variant, natural gene therapy

Due to a production error, an incorrect Data Availability Statement was provided. The correct statement is “The original contributions presented in the study are publicly available. This data can be found here: https://www.ncbi.nlm.nih.gov/bioproject/PRJNA1013760”.

The publisher apologizes for this mistake. The original version of this article has been updated.

